# In Thickness and in Health: Delayed-Onset Pompe Disease Resembling Hypertrophic Cardiomyopathy

**DOI:** 10.7759/cureus.92598

**Published:** 2025-09-17

**Authors:** Glenmore Lasam, Maria Kristina Cassandra Lasam

**Affiliations:** 1 Cardiovascular Disease, Stockton Cardiology Medical Group, Manteca, USA; 2 Biomedical Pathway, Mountain House High School, Mountain House, USA

**Keywords:** acid alpha-glucosidase deficiency, cardiac myosin inhibitor, hypertrophic cardiomyopathy, left ventricular outflow tract obstruction, pompe disease, septal myectomy

## Abstract

Delayed-onset Pompe disease is an extremely rare genetic disease in adults and should be considered in patients with severe left ventricular hypertrophy with left ventricular outflow tract (LVOT) obstruction due to its resemblance to hypertrophic cardiomyopathy (HCM). A case of a 58-year-old woman with no known comorbidities with excellent functional capacity presented because of the gradual onset of exertional dyspnea associated with fatigue, diminished exercise tolerance, occasional exertional chest tightness, and lightheadedness. The electrocardiogram showed left ventricular hypertrophy with T wave inversion in the anterolateral leads. Transthoracic echocardiogram and cardiac magnetic resonance (CMR) showed HCM with LVOT obstruction. Nuclear cardiac scintigraphy was unremarkable for transthyretin cardiac amyloidosis. The patient received a beta blocker and cardiac myosin inhibitor but experienced no change in symptoms. She had a ventriculomyotomy-myectomy and mitral valve repair, which significantly improved her symptoms and resolved the LVOT obstruction. Histology revealed myocyte hypertrophy and both interstitial and endocardial fibrosis. Genetic testing detected a pathogenic c.1841C>T (p.Thr614Met) variant in the acid alpha-glucosidase (GAA) gene linked to autosomal recessive Pompe disease. She is being followed as an outpatient and reports marked improvement in exertional dyspnea and exercise tolerance. She has been referred to a specialist center for enzyme replacement.

## Introduction

Pompe disease is a rare genetic disorder due to acid alpha-glucosidase (GAA, also called acid maltase) deficiency and was the first identified lysosomal storage disease categorized as glycogen storage disease type II (GSD II). The estimated incidence was one in 40,000 in a newborn screening study [1]. This autosomal-recessive disorder stems from pathogenic variants in the GAA gene located at 17q25.2-q25.3, with over 560 identified variants [2]. The deficiency of the enzyme results in accumulation of glycogen in lysosomes and in the cytoplasm, causing tissue destruction [3], and may expand to vesicle systems that are connected to lysosomes, which can influence receptors, such as glucose transporter 4, that sequence through these organelles [4]. There has been a paucity of information in the literature regarding delayed-onset Pompe disease resembling hypertrophic cardiomyopathy (HCM). This case illustrates the need to investigate alternative causes of severe left ventricular hypertrophy to establish appropriate management promptly. Though rare in adults, Pompe disease should be considered in patients with left ventricular outflow tract (LVOT) obstruction due to its resemblance to HCM. 

This article was previously presented as a poster presentation at the American College of Cardiology 74^th^ Annual Scientific Session on March 29, 2025, in Chicago, Illinois, USA.

## Case presentation

**Figure 1:**
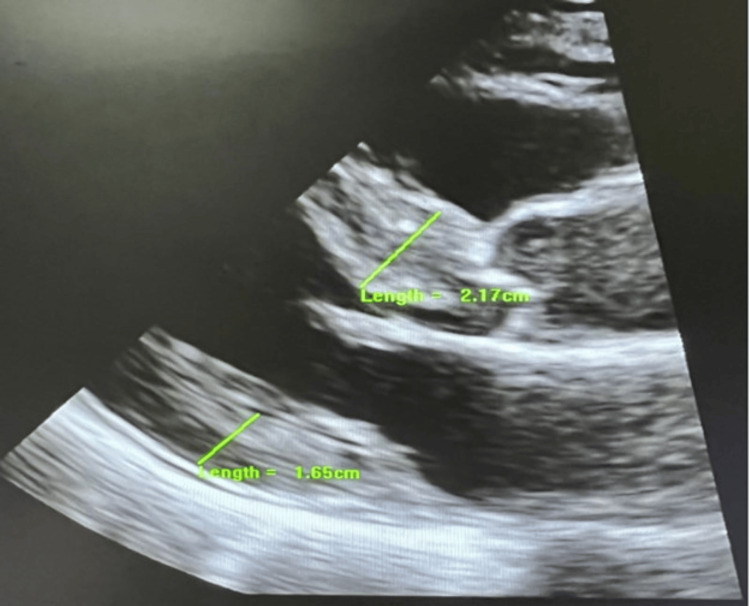
Transthoracic echocardiogram in a parasternal view showed severe left ventricular hypertrophy, which measures 2.17 cm in the basal septum and 1.65 cm in the basal left ventricular posterior wall.

**Figure 2:**
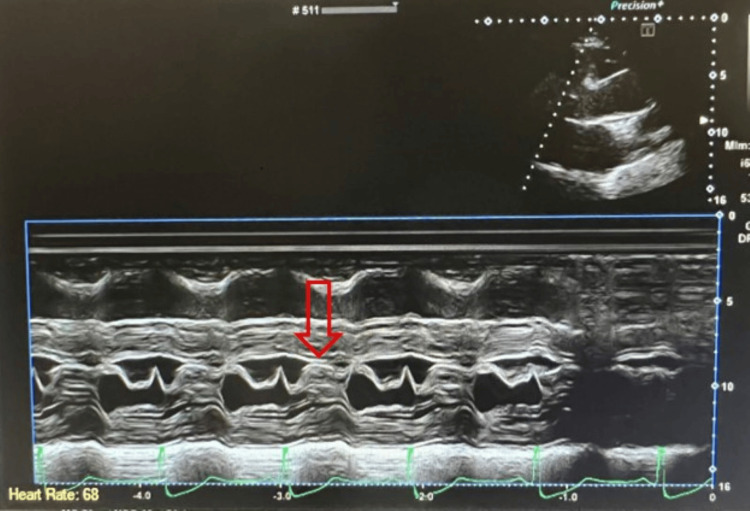
Transthoracic echocardiogram in M-mode showed systolic anterior motion of the anterior leaflet of the mitral valve (red arrow).

**Figure 3:**
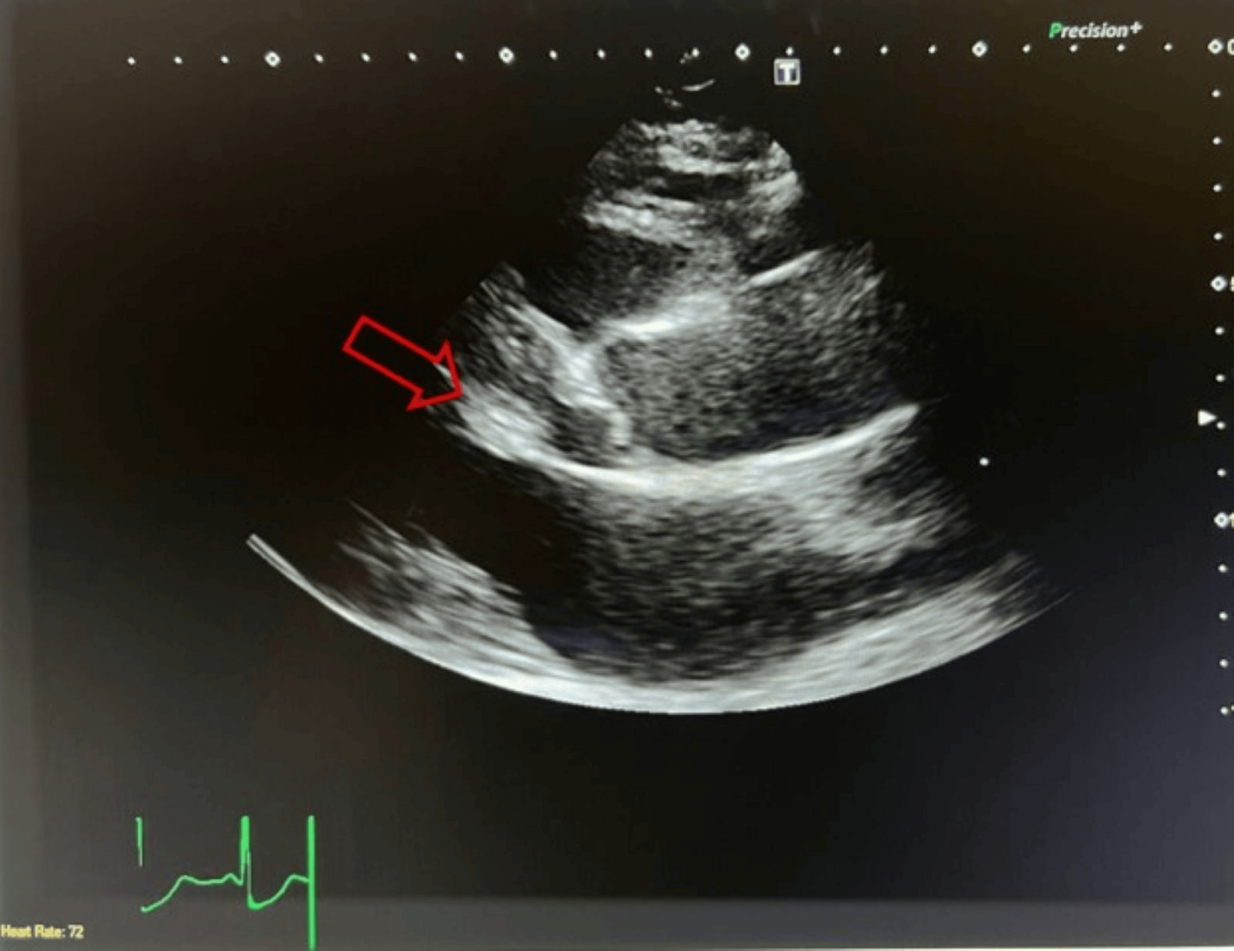
Transthoracic echocardiogram in a parasternal view showed systolic anterior motion of the anterior leaflet of the mitral valve (red arrow).

**Figure 4:**
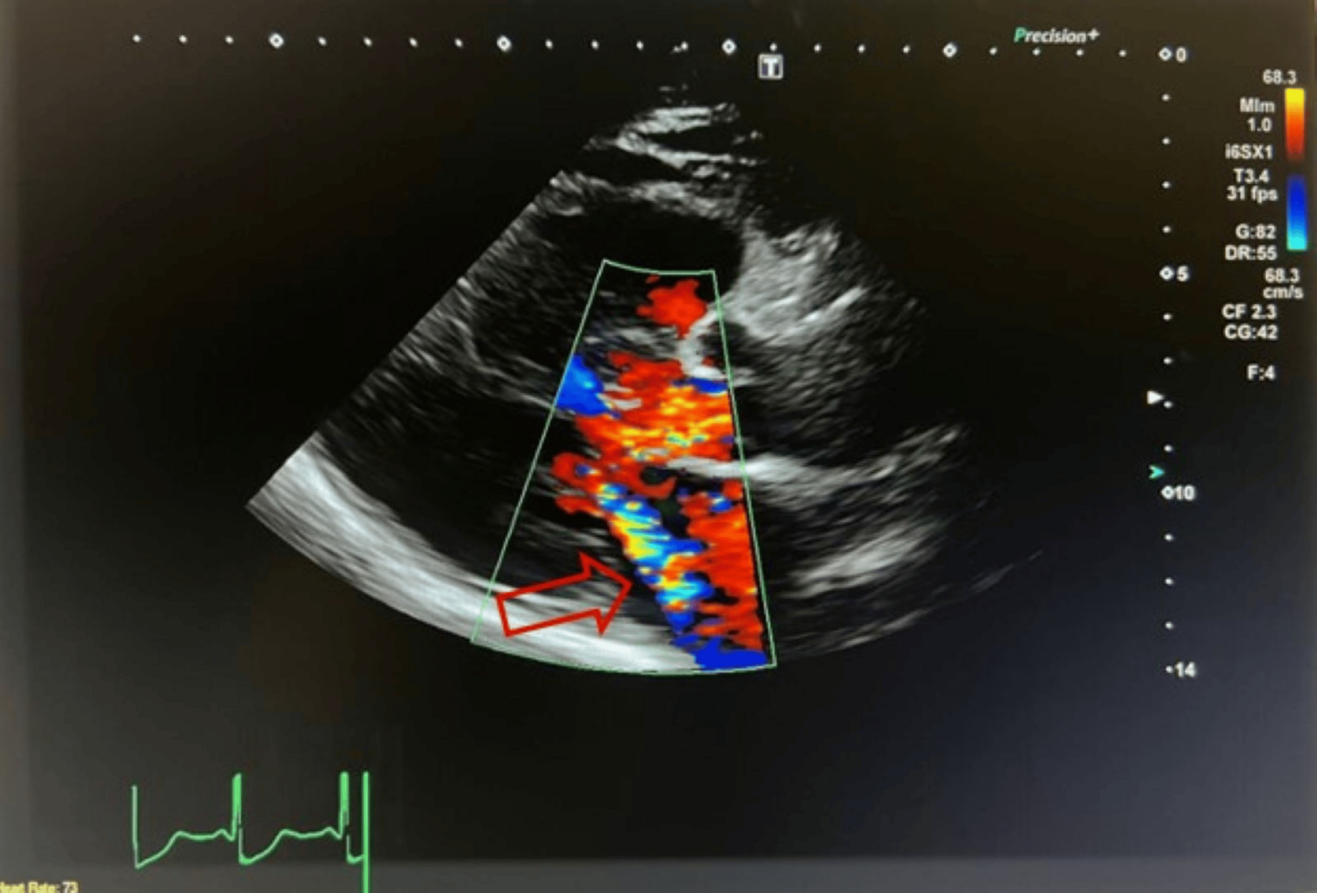
Transthoracic echocardiogram in a parasternal view showed posteriorly directed mitral regurgitation, typically seen in severe left ventricular hypertrophy with systolic anterior motion of the anterior leaflet of the mitral valve (red arrow).

**Figure 5:**
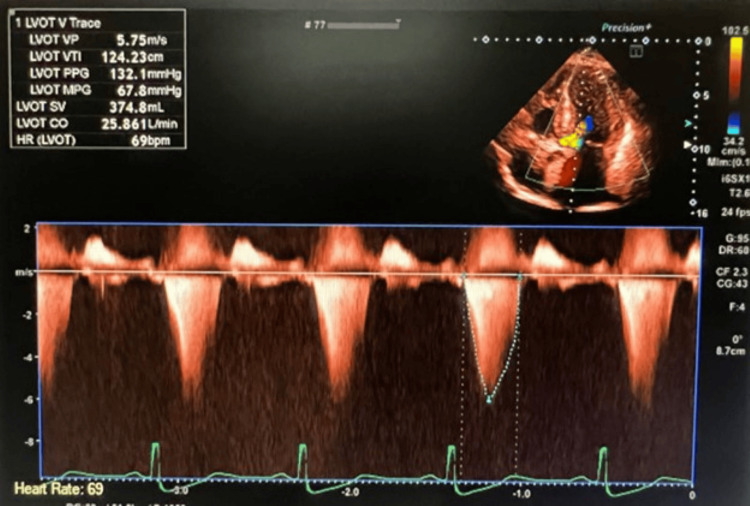
Transthoracic echocardiogram with a pulsed wave Doppler of the LVOT in a five-chamber view pre-ventriculomyotomy-myectomy showed a resting peak gradient of 132.1 mmHg, which was significantly elevated.

**Figure 6:**
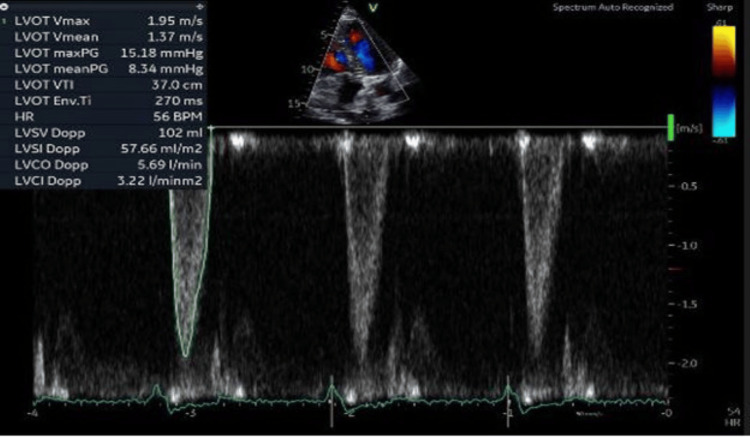
Transthoracic echocardiogram with a pulsed wave Doppler of the LVOT in a five-chamber view post ventriculomyotomy-myectomy with mitral valve repair showed a normal resting peak gradient of 15.18 mmHg.

A healthy 58-year-old woman with no known comorbidities presented to the clinic because of exertional dyspnea. She endorsed that she developed a gradual onset of worsening exertional dyspnea for the past 12 months associated with fatigue and diminished exercise tolerance. Prior to the emergence of her symptoms, she had an excellent functional capacity, described as being able to hike more than 25 miles in the mountains and run half marathons without issues. She noticed a progression of her symptoms for the last couple of months, and recently she had trouble walking short distances and climbing up a flight of stairs. She also endorsed occasional exertional chest tightness and lightheadedness that resolve with rest. She denied any history of syncope, heart attack, stroke, or cardiac procedures. She was not aware of any HCM, sudden cardiac deaths, or premature coronary artery disease in the family. She is not an active smoker, alcohol drinker, or illicit drug user. The initial physical examination revealed a middle-aged woman with difficulty breathing on brisk ambulation but not in distress. Vital signs showed a temperature of 98.5℉, blood pressure of 110/70 mmHg, heart rate of 88 beats per minute (bpm), respiratory rate of 18, and oxygen saturation of 98% on room air. Cardiac examination revealed regular rhythm with a mid-systolic crescendo-decrescendo murmur at the left sternal border radiating to the apex. Respiratory assessment elucidated clear breath sounds. She had no swelling or cyanosis in her lower extremities. The electrocardiogram showed sinus rhythm, left ventricular hypertrophy, and T wave inversion in the anterolateral leads. The initial transthoracic echocardiogram showed preserved left ventricular systolic function with an ejection fraction of 65%, hypertrophic cardiomyopathy with a systolic anterior motion of the anterior leaflet of the mitral valve (Figures 1-3), posteriorly directed mitral regurgitation (Figure 4), and an elevated resting LVOT gradient of 132.1 mmHg (Figure 5). Cardiac magnetic resonance (CMR) imaging revealed HCM with restriction of flow in the LVOT during systole. Nuclear cardiac scintigraphy with pyrophosphate scan was negative for transthyretin cardiac amyloidosis. The patient was started on a beta blocker and cardiac myosin inhibitor with no improvement in her symptoms. She underwent ventriculomyotomy-myectomy with mitral valve repair. LVOT resting gradient normalized to 15.18 mmHg (Figure 6), and her symptoms improved significantly. Histology showed myocyte hypertrophy with interstitial and endocardial fibrosis. Genetic testing revealed a pathogenic variant, c.1841C>T(p.Thr614Met), identified in the GAA gene associated with autosomal recessive Pompe disease. She has been followed up as an outpatient and has been feeling better with significant improvement of exertional dyspnea and exercise tolerance. She was referred to a specialized center for consideration of enzyme replacement. She has been closely monitored for any recurrence of exertional dyspnea, fatigue, and diminished exercise tolerance, as well as for evidence of an elevated LVOT gradient, which would warrant the immediate initiation of enzyme replacement therapy.

## Discussion

Pompe disease is a rare genetic disorder caused by GAA deficiency, resulting in lysosomal glycogen buildup in all tissues. The enzyme activity is linked to genotype, being minimal or absent in infantile-onset patients and reduced in late-onset patients [5]. It has an infantile-onset ("classic") form presenting with HCM and a late-onset form (including juvenile and adult presentations) that generally lacks cardiac symptoms [6]. Elevated serum creatine kinase (CK) and reduced leukocyte GAA activity are typical indicators of the GAA deficiency [7]. Muscle biopsy shows vacuolar myopathy with glycogen stored in lysosomes and free in the cytoplasm [8]. Infantile-onset GAA deficiency usually presents symptoms appearing at around four months, which include cardiomegaly, respiratory compromise, muscular hypotonia, feeding issues, and failure to thrive [9]. Late-onset GAA deficiency patients may develop skeletal myopathy at any age, leading to respiratory failure in the second or third decade of life, but do not develop cardiomyopathy [10]. A multinational survey of individuals aged 2.6 to 81 years with late-onset GAA deficiency showed the age at first complaint ranged from 0 to 62 years, and diagnosis age from 0 to 66 years [11]. The measurement of GAA enzyme activity serves as a dependable diagnostic method for identifying all types of GAA deficiency. Gene sequencing is commonly used to confirm diagnosis because it is routinely accessible, minimally invasive, provides genotype-phenotype information, and can predict cross-reactive immunologic material status in some cases [7]. The C.-13-13T.G splice mutation is the most prevalent variant in late-onset GAA deficiency, with an allele frequency of 40% to 70% [12]. If an individual is homozygous for c.1841C>T(p.Thr614Met) and has clinical signs consistent with Pompe disease, as in our case, then this variant is quite strong evidence toward a diagnosis of delayed‑onset Pompe disease. Using a muscle biopsy to measure GAA activity can confirm the diagnosis; however, it involves invasiveness, anesthesia, and risks of false positives from poor sample processing.

The other etiologies for late-onset diseases that present with HCM include metabolic disorders such as GSD type V (McArdle disease), GSD type VI, and Fabry disease, as well as muscular dystrophies, including Duchenne-Becker muscular dystrophy and limb-girdle muscular dystrophy. These diseases have been ruled out in this case by the result of genetic testing. Although our patient has significant HCM as seen in echocardiogram and CMR, her genetic testing did not reveal the classic HCM gene mutation. Transthyretin cardiac amyloidosis was also unlikely due to unremarkable cardiac pyrophosphate scintigraphy and genetic testing, as well as the absence of abnormal contrast kinetics on CMR. An unusual adult-onset Pompe disease has been documented that initially presented as cerebral stroke and was accompanied by left ventricular hypertrophy on echocardiogram [13]. In another study to evaluate the prevalence of cardiovascular abnormalities in eighty-seven patients with late-onset Pompe disease, four patients, or 5%, had a mildly elevated left ventricular mass index with a range of 92-97 g/m² in three women and 104 g/m² in a man [14]. A thorough search of PubMed (National Library of Medicine), the Cochrane Library (Cochrane Database of Systematic Reviews), and Online Mendelian Inheritance in Man (OMIM), utilizing keywords including "Pompe disease" and "HCM" with the use of inclusion criteria comprising adult onset, myectomy, beta blocker, and cardiac myosin inhibitor, revealed no documented cases.

A multidisciplinary care team is essential in the coordination of care, which includes a geneticist, physiatrist, speech therapist, cardiologist, pulmonologist, orthopedist, and nutritionist. Many patients with late-onset cases require noninvasive ventilation during sleep to manage nocturnal hypoxemia and diurnal hypercapnia, and in some instances, mechanical ventilation may eventually become necessary. Enzyme replacement therapy (ERT) is the main treatment for GAA deficiency. Alglucosidase alfa significantly reduced the risk of death by 95% and invasive ventilation by 91% and improved cardiomyopathy and motor skills [15]. Institution of ERT as early as 18 hours of age resulted in resolution of HCM with normal neurodevelopmental outcomes at 46 weeks [16]. Although evidence is limited, data indicate that patients with juvenile-onset GAA deficiency (symptoms starting between ages two and 18) may benefit from ERT, showing improved muscle strength and diminished demand for assisted ventilation [17]. In patients with hypertrophic obstructive cardiomyopathy (HOCM), maximal medical management primarily using beta blockers or calcium channel blockers has been the first palliative approach and generally more effective for patients with mild to moderate symptoms [18]. Recently, the cardiac myosin inhibitor mavacamten was added to the medical regimen, with 37% of patients showing at least a one-class New York Heart Association (NYHA) symptom improvement and modest gains in peak oxygen consumption versus placebo [19]. Septal myectomy is used in Pompe disease and HCM patients with LVOT obstruction, causing symptoms like shortness of breath, chest pain, or dizziness, since it relieves obstruction to improve exercise and daily activity, but does not cure Pompe disease or HCM. Amelioration in heart failure symptoms is observed in more than 90% of patients, as indicated by at least a one-class improvement in NYHA functional class, with 75% becoming asymptomatic, transitioning from NYHA class III/IV to class I. No significant outcome differences are reported between men and women, and ongoing postoperative drug treatment is not required [18]. Alcohol septal ablation is typically used for older patients, those with major comorbidities, or when myectomy poses excessive risk or is unavailable [20].

## Conclusions

This case demonstrates the importance of investigating other etiologies of severe left ventricular hypertrophy to institute definitive management in a timely manner. Although Pompe disease is a rare occurrence, especially in adults, it should be considered in patients presenting with clinical manifestations of LVOT obstruction since it can resemble HCM. This case highlighted the significance of having that in the list of differential diagnoses in an adult patient presenting with worsening exertional dyspnea, fatigue, and diminished exercise capacity.
